# Viral Live‐Attenuated Vaccines (LAVs): Past and Future Directions

**DOI:** 10.1002/advs.202407241

**Published:** 2024-12-06

**Authors:** Yan‐Dong Tang, Yuming Li, Xue‐Hui Cai, Xin Yin

**Affiliations:** ^1^ State Key Laboratory for Animal Disease Control and Prevention Harbin Veterinary Research Institute of Chinese Academy of Agricultural Sciences Harbin 150069 China; ^2^ Heilongjiang Provincial Research Center for Veterinary Biomedicine Harbin Veterinary Research Institute of Chinese Academy of Agricultural Sciences Harbin 150069 China; ^3^ Heilongjiang Provincial Key Laboratory of Veterinary Immunology Harbin Veterinary Research Institute of Chinese Academy of Agricultural Sciences Harbin 150069 China; ^4^ School of Public Health Shandong First Medical University & Shandong Academy of Medical Sciences Ji'nan 250117 China; ^5^ Key Laboratory of Emerging Infectious Diseases in Universities of Shandong Shandong First Medical University & Shandong Academy of Medical Sciences Tai'an 271000 China

**Keywords:** adaptation, attenuation, evolution, live attenuated vaccines (LAVs), pathogenicity

## Abstract

Viral infections continue to pose a significant threat to the health of both humans and animals. Currently, live attenuated vaccines (LAVs) remain the most efficacious and widely utilized tool for combating viral infections. Conventional LAVs involve the adaptation of virulent viruses to novel hosts, cell cultures, or suboptimal environments, resulting in a reduction in pathogenicity while retaining immunogenicity. This process entails directed evolution of the virus to enhance its replication efficiency under these modified conditions. In this review, the development of traditional animal‐adapted and cold‐adapted LAVs is specially discussed. Additionally, the factors that contribute to virus attenuation from a viral lifecycle perspective are summarized. Finally, we propose future directions for next‐generation LAVs.

## Graphical abstract

Live attenuated vaccines represent a delicate equilibrium between virulence and immunogenicity during adaptation. The process of animal adaptation involves artificial selection; therefore, adapted viruses exhibit decreased pathogenicity to their original hosts and increased pathogenicity to their adapted hosts. Highly adapted rinderpest virus (RPV) gradually acquires pathogenic adapted animals and loses its pathogenicity for cattle.

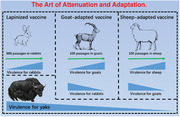

## 1 Live Attenuated Vaccines (LAVs) Against Viruses

Infectious diseases are a significant threat to humans and animals; historically, human deaths caused by infectious diseases have even surpassed those caused by wars.^[^ ^1^ ^]^ Bacterial infectious diseases are well controlled by the development of antibiotics, but viral diseases remain a significant challenge to our society. For instance, severe acute respiratory syndrome coronavirus 2 (SARS‐CoV‐2), Middle East respiratory syndrome coronavirus (MERS‐CoV), Ebola virus, and emerging and re‐emerging influenza viruses have been identified in recent years.^[^ ^2^, ^3^ ^]^ Vaccination is a cornerstone of public health policy, and live attenuated vaccines (LAVs) undoubtedly represent the most successful tool to date to combat viral diseases.^[^ ^4^, ^5^ ^]^ The advantage of using LAVs lies in their ability to mimic natural infection by replicating within host cells and stimulating both humoral and cellular immune responses. These vaccines not only prevent immediate viral infections but also provide long‐lasting immunity. Traditionally, the development of LAVs has involved adapting highly pathogenic viruses to cell culture, novel hosts, or suboptimal environments, followed by reducing their virulence while retaining immunogenicity to elicit a robust protective immune response. In fact, this adaptation process of a virus is a directed evolution of the virus, and ideal LAVs represent a delicate equilibrium between virulence and immunogenicity. This process is inherently time‐consuming, but advancements in molecular biology have led to the development of innovative approaches for rapid viral attenuation.^[^ ^3^, ^6^ ^]^ Traditional LAVs, such as those against smallpox, polio, yellow fever, measles, mumps, and rotavirus, are still the most effective and most‐used vaccines.^[^ ^5^ ^]^ Most of these LAVs were developed to adapt pathogenic viruses to cell culture and have been well reviewed; therefore, these LAVs are not discussed here.^[^ ^5^ ^]^ In this review, we will highlight the development of several unique LAVs, for example, the attenuation of viruses via animal adaptation or cold adaptation. Furthermore, during the process of viral attenuation, the immunogenicity of the virus is a critical determinant for LAVs. The level of immunogenicity plays a pivotal role in assessing the suitability of an attenuated virus as a vaccine. Last, we summarize how virulent viruses are attenuated from the view of the viral lifecycle and provide potential future directions for the next generation of LAVs.

### 1.1 Animal‐Adapted LAVs

**Figure 1:**
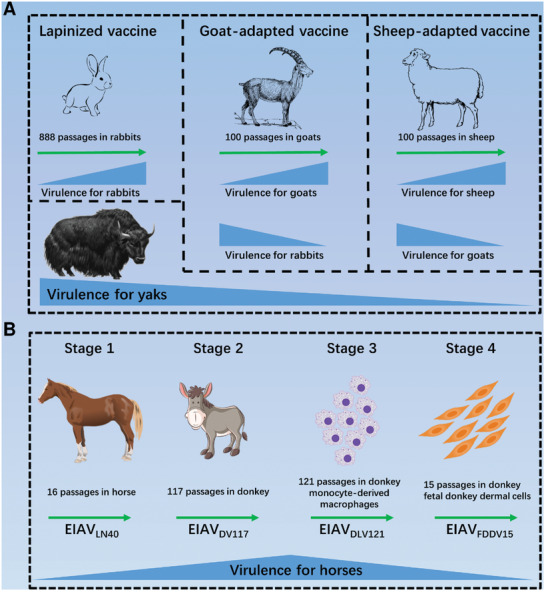
Animal‐adapted strategy for the development of LAVs. A) The development of Rinderpest LAVs: Virulent RPV underwent 888 serial passages in rabbits, resulting in a lapinized vaccine. The lapinized RPV strain was then subjected to 100 serial passages in goats, leading to a goat‐adapted vaccine. Subsequently, the goat‐adapted virus was further adapted to sheep for 100 passages, ultimately generating a sheep‐adapted vaccine. B) The development of EIAV LAVs: EIAV_LN40_ was isolated and subsequently passaged 16 times in horses. Next, EIAV_LN40_ was subjected to 117 passages (EIAV_DV117_) in donkeys. EIAV_DV117_ was further cultured in dMDMs for 121 passages (EIAV_DLV121_). Finally, EIAV_DLV121_ was adapted to fetal donkey dermal cells for 13 passages (EIAV_FDDV13_). (https://openclipart.org/).

Animal‐adapted LAVs are mainly generated in veterinary fields, such as vaccines against rinderpest virus (RPV), classical swine fever virus (CSFV), and equine infectious anemia virus (EIAV).^[^ ^7^, ^8^, ^9^ ^]^ RPV, which belongs to the *Morbillivirus* genus within the *Paramyxoviridae* family, is one of the most devastating bovine viruses.^[^ ^10^ ^]^ Rinderpest was the second disease to be globally eliminated after smallpox; this achievement potentially renders the RPV vaccine the most successful LAV as the smallpox vaccine was not derived from *Variola* and therefore cannot be strictly classified as an attenuated vaccine.^[^ ^5^ ^]^ The Rinderpest virus was eradicated in China as early as 1956.^[^ ^11^ ^]^ The Nakamura III vaccine strain, also known as the lapinized vaccine, was generated via serial passages of RPV in rabbits and represents the earliest development of RPV vaccines in China.^[^ ^7^, ^11^ ^]^ However, this vaccine was inadequate for addressing all RPV issues in China. For example, the yield of this vaccine is low, and it poses safety risks in Korean cattle and yaks, leading to severe adverse effects or even fatality.^[^ ^11^ ^]^ To overcome these problems, scientists have attempted to adapt this lapinized RPV strain (888 passages in rabbits) to goats (**Figure** **1**A). After 100 in vivo passages in goats, a goat‐adapted vaccine was finally developed. A single goat can produce up to 5000 doses of this vaccine; however, the use of a goat‐adapted vaccine also results in a mortality rate of 17.5% among vaccinated yaks.^[^ ^11^ ^]^ To develop a safer yak vaccine, a goat‐adapted virus was further adapted in sheep for 100 passages. Eventually, a sheep‐adapted RPV vaccine was successfully developed in 1952, and this vaccine successfully addressed safety issues in Korean cattle and yaks.^[^ ^11^ ^]^ In China, with the wide use of sheep‐adapted RPV vaccines, the Rinderpest has been completely eradicated.

Viruses can therefore be attenuated in novel hosts. Based on this experience, CSFV vaccines have also been developed by the in vivo serial passaging of classical swine fever virus in rabbits.^[^ ^12^ ^]^ However, importantly, the applicability of this experience varies among different viruses, such as EIAV, a member of the *Retroviridae* family. The EIAV vaccine is distinctive because it incorporates adaptations of EIAV to both animals and cell culture.^[^ ^13^ ^]^ This vaccine originated from a moderately pathogenic strain, EIAV_LN40,_ which was isolated in Liaoning Province, China. EIAV_LN40_ was subsequently passaged 16 times in horses to acquire a highly pathogenic strain that was 100% lethal to horses (Figure 1B). Many attempts have been made to attenuate EIAV_LN40_ by serially passaging it through heterologous animals. However, the host range of EIAV is limited, and these attempts have failed because EIAV was unable to infect most of the tested animals. Ultimately, EIAV_LN40_ was identified to exhibit low pathogenicity toward donkeys. Scientists speculated that adapting EIAV_LN40_ to donkeys may attenuate its virulence in horses. However, after 117 (EIAV_DV117_) in vivo passages in donkeys, EIAV_DV117_ evolved into a strain supervirulent to horses and donkeys instead of being attenuated.^[^ ^14^ ^]^ Fortunately, at that point, scientists were able to successfully cultivate primary donkey monocyte‐derived macrophages (dMDMs) in vitro. They then further attempted to adapt EIAV_DV117_ in dMDMs; after 121 passages (EIAV_DLV121_), EIAV was attenuated and no longer manifested any clinical symptoms in donkeys or horses.^[^ ^15^ ^]^ Because primary dMDMs may potentially be contaminated, scientists have attempted to adapt EIAV_DLV121_ to continuous cell lines known as fetal donkey dermal (FDD) cells. After 13 passages, a vaccine specifically adapted to fetal donkey dermal cells (EIAV_FDDV13_) was developed. Since then, EIAV_DLV121_ and EIAV_FDDV13_ have been extensively applied in China for controlling the EIAV epidemic. This vaccine is the only vaccine extensively used to control lentivirus; in contrast, despite its similarity to EIAV, no commercially available vaccine has yet been developed for HIV‐1.

In general, during the adaptation of viruses to heterologous animals, viruses gradually evolve to adapt to new hosts. As shown in Figure 1A, viruses exhibit decreased pathogenicity to their original hosts and increased pathogenicity to their adapted hosts. For example, RPV isolated from cattle is nonpathogenic to rabbits; however, after in vivo adaptation, infected rabbits gradually develop fever, and highly adapted RPV strains exhibit high lethality to rabbits. The pathogenicity of lapinized RPV toward goats increases during adaptation in goats, while its virulence is attenuated in rabbits.^[^ ^11^ ^]^ Another example is the lapinized CSFV vaccine, in which CSFV gradually increased its pathogenicity toward rabbits but lost its pathogenicity toward pigs during in vivo passages in rabbits.^[^ ^12^ ^]^ However, importantly, this does not imply a continuous increase in virulence toward novel hosts during adaptation. In the development of the aforementioned vaccines, viral passages were under deliberate selection to enhance viral adaptability. For instance, RPV and CSFV, which are viruses that induce persistent fever in rabbits, were specifically chosen for next‐generation passaging. Notably, viral mutations can occur randomly during adaptation, resulting in various phenotypes exhibiting either increased or decreased virulence. The process of animal adaptation involves artificial selection, which leads to increased virulence toward novel hosts. Adapting a virus to different animals can be challenging due to the complex interactions that govern the host range of a virus, including receptor binding, co‐opting of cellular machinery, and evasion of innate immunity.^[^ ^16^ ^]^ During adaptation, viruses evolve to adapt to novel hosts by overcoming these species‐specific restrictions and gradually increasing their pathogenicity to the adapted hosts. Moreover, adapted viruses gradually lose their virulence to their original host. Interestingly, the pathogenicity of EIAV to horses was enhanced through serial passaging in donkeys, possibly due to the close genetic relationship between horses and donkeys. It is imperative to emphasize that although a virus is attenuated, this does not necessarily mean that it can be used as a vaccine. Because it cannot be guaranteed that attenuated viruses will induce a protective immune response, immunogenicity is another key factor for vaccines. Immunogenicity plays a crucial role in determining whether an attenuated virus can be utilized as a vaccine. Conferring long‐lasting immunity against the targeted pathogen is the desired outcome of vaccination. Therefore, extensive studies must be conducted to evaluate the immunogenicity of attenuated viruses. For instance, several teams have attempted to develop an EIAV vaccine by passaging the virus in horse or donkey MDMs; however, although some strains were successfully attenuated, they failed to elicit a protective immune response in horses. Among these attempts, only EIAV_DLV121_ was able to induce a protective immune response, which may be attributed to its excellent immunogenicity. Therefore, an effective LAV must not only involve reduction in the virulence of a virus but also preserve its original immunogenicity.

### 1.2 Cold‐Adapted LAVs

The cold‐adapted strategy attenuates a wide range of viruses, including *Enterovirus*, *Flavivirus*, *Arenavirus*, *Paramyxovirus*, *Rhabdovirus*, *Herpesvirus*, and *Poxvirus*.^[^ ^17^ ^]^ The most successful example is the live attenuated influenza vaccine (LAIV).^[^ ^18^ ^]^ Since the 1930s, LAIVs have been developed through successive passages of influenza virus in alternative hosts, such as ferrets, mice, or chicken embryos.^[^ ^19^ ^]^ Nevertheless, these methods have limitations, such as suboptimal efficacy, safety concerns, and low yield.^[^ ^19^ ^]^ In the 1960s, the concept of cold‐adapted LAIVs was proposed to attenuate influenza viruses through adapting the virus to embryonated eggs at progressively decreasing temperatures (36–25 °C), through which the virus undergoes evolutionary changes and gradually adapts to low temperatures while losing its ability to replicate at high temperatures (37 °C).^[^ ^19^, ^20^ ^]^ Consequently, when administered via inhalation immunization, this vaccine replicates effectively in the nasal cavity where temperatures are lower (e.g., 25 °C); it subsequently stimulates mucosal and cellular immunity.^[^ ^21^ ^]^ Furthermore, cold‐adapted viruses exhibit limited replication capacity in the lungs at 37 °C and display temperature sensitivity, thereby ensuring the safety of these viruses.^[^ ^18^ ^]^ In fact, the most effective defense against viral infections occurs at mucosal surfaces, which serve as entry points for viruses. Secretory immunoglobulin A (IgA) plays a pivotal role in neutralizing pathogens and facilitating their clearance at mucosal surfaces.^[^ ^22^ ^]^ In the case of influenza virus, only intranasal immunization induces the production of protective secretory IgA.^[^ ^23^ ^]^ Thus, from this perspective, cold‐adapted vaccines are particularly suitable for treating respiratory viruses. In fact, this strategy has been applied for SARS‐CoV‐2 and MERS‐CoV vaccines in recent years.^[^ ^24^, ^25^ ^]^

## 2 Mechanisms of Conventional Attenuation

**Figure 2:**
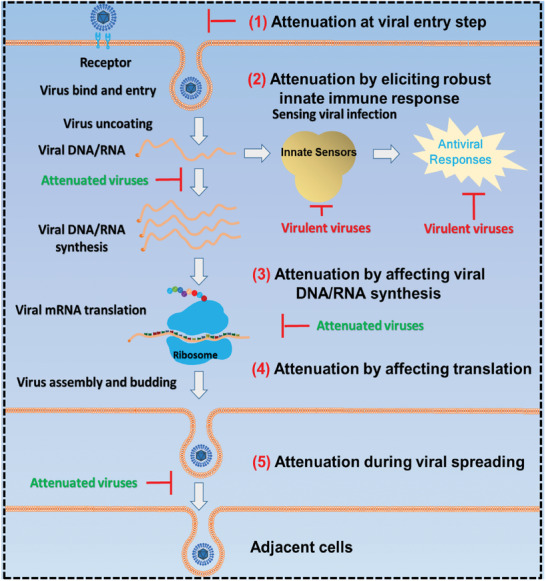
Attenuation mechanisms of viruses. Despite various strategies employed to develop LAVs, abnormalities in the viral lifecycle occur during the replication of LAVs within host cells. Here, we summarize the means by which a virus undergoes attenuation through adaptation during its life cycle. The images used in this figure were generated by the authors.

The lifecycle of a virus includes the cellular entry of a virus, synthesis of viral RNA/DNA, translation, and release. Although distinct strategies have been used to develop LAVs, it is evident that some aberrations occur in the viral lifecycle during the replication of attenuated viruses in vivo. Herein, we summarize how a virus is attenuated during adaptation from the perspective of its viral life cycle (**Figure** **2**).

### 2.1 Attenuation at the Viral Entry Step

Viral attachment and entry are the initial steps of viral infection. When viral entry goes wrong, pathogenicity may be attenuated. A first example is the yellow fever virus (YFV) vaccine 17D, which was developed by serial passaging of the wild‐type Asibi strain in mouse and chicken embryonic tissues. The 17D genome has multiple mutations, predominantly within the envelope (E) protein.^[^ ^26^ ^]^ The Asibi strain infects HeLa cells through classic clathrin‐mediated endocytosis, while YFV‐17D enters host cells through an alternative clathrin‐independent pathway.^[^ ^27^, ^28^ ^]^ The attenuations of RPV and measles virus (MeV) are also correlated with the cellular entries of these viruses. MeV is genetically closely related to RPV, and its divergence occurred in the sixth century BCE.^[^ ^29^ ^]^ The hemagglutinin (H) gene is considered a critical factor in determining interspecies infectivity, and the H gene was identified as a determinant of pathogenicity in a rabbit model of RPV infection.^[^ ^30^ ^]^ LAVs against MeV were developed by adapting the MeV Edmonston strain to propagate in a variety of cell lines.^[^ ^31^ ^]^ The attenuated MeV phenotype may be attributed to multiple mutations in distinct genes; however, the most prominent change observed in entry receptor tropism is also determined by the presence of the viral H gene.^[^ ^32^ ^]^ Pathogenic MeV strains exclusively utilize signaling lymphocyte activation molecule (SLAM F1, also known as CD150) and nectin‐4 as entry receptors. However, tissue‐ or cell‐culture‐adapted strains use CD46 or other identified receptors for cellular entry.^[^ ^32^ ^]^ The attenuations of other viruses, such as West Nile virus (WNV), Japanese encephalitis virus (JEV), and Chikungunya virus (CHIKV), are also correlated with cellular entry mechanisms.^[^ ^33^, ^34^ ^]^

### 2.2 Attenuation by Eliciting a Robust Innate Immune Response

After the virus enters cells, it is uncoated and releases viral nucleic acids. During this step, innate immune sensors sense and recognize the viral nucleic acid, triggering antiviral responses to combat viral infection.^[^ ^35^ ^]^ The innate immune response also plays a pivotal role in initiating adaptive immunity against viral infections.^[^ ^36^ ^]^ Interestingly, to replicate efficiently, viruses have evolved various strategies to evade the host innate immune system.^[^ ^37^ ^]^ Therefore, when viruses lose their ability to counteract innate immunity, their pathogenicity is attenuated. For example, virulent MeV and YFV block the production of type I interferon (IFN‐α and IFN‐β), while vaccine strains of MeV and YFV effectively stimulate the production of type I IFN.^[^ ^27^, ^28^, ^38^ ^]^ The nonstructural 1 (NS1) influenza virus protein functions to counteract the host type I IFN response, and NS1‐truncated influenza viruses exhibit an attenuated phenotype.^[^ ^39^, ^40^ ^]^ The African swine fever virus (ASFV) contains several viral genes that counteract the type I IFN response, and some potential LAV candidates against ASFV have been developed by deleting these genes.^[^ ^41^ ^]^ Several other viruses, including SARS‐CoV‐2,^[^ ^42^ ^]^ rabies virus (RV),^[^ ^43^ ^]^ pseudorabies virus (PRV),^[^ ^44^ ^]^ and porcine reproductive and respiratory syndrome virus (PRRSV),^[^ ^45^ ^]^ are also attenuated via the modification of viral genes that counteract innate immunity. Therefore, understanding how viruses evade innate immunity facilitates the rational design of attenuated vaccines.

### 2.3 Attenuation by Modulating Viral RNA/DNA Synthesis

The synthesis of viral nucleic acids is a crucial step for the replication of both RNA and DNA viruses. The cold‐adapted influenza vaccine is a representative virus that is attenuated through the modulation of viral RNA synthesis. There are three distinct phenotypes for this vaccine: cold adaptation (*ca*), temperature sensitivity (*ts*), and attenuation (*att*), which are associated with viral RNA synthesis.^[^ ^46^ ^]^ The viral RNA synthesis of influenza virus requires a heterotrimeric viral RNA‐dependent RNA polymerase (RdRp), which is composed of polymerase basic 1 (PB1), polymerase basic 2 (PB2), and polymerase acidic (PA). These components bind to viral genomic RNA and form a complex, also known as viral ribonucleoproteins (vRNPs).^[^ ^47^ ^]^ vRNPs are responsible for viral gene transcription and viral RNA synthesis. Cold‐adapted mutations in vRNPs only promote viral RNA synthesis at lower temperatures (e.g., 25 °C) but impair viral RNA synthesis at higher temperatures, such as 37 °C.^[^ ^46^, ^48^ ^]^ In fact, viral RNA synthesis activity is closely associated with virulence. For instance, in the case of PRRSV, nonstructural protein 9 (Nsp9, an RdRp for PRRSV) regulates viral pathogenicity through its ability to synthesize viral RNA.^[^ ^49^, ^50^ ^]^ In a CSFV LAV, nonstructural protein 4B (NS4B) contributes to its attenuation phenotype by decreasing viral RNA synthesis.^[^ ^51^ ^]^ Notably, viral RNA synthesis activity is correlated with virulence exclusively in host target cells rather than in adapted cell lines. For example, the replication kinetics of cell line‐adapted LAVs consistently surpass those of virulent strains in the adapted cell line; however, they replicate less efficiently in hosts or within host primary cells.

For DNA viruses or lentiviruses (retroviruses), which have a DNA synthesis stage, the in vivo target cells are often terminally differentiated cells characterized by low levels of deoxynucleoside triphosphates (dNTPs).^[^ ^52^ ^]^ DNA viruses, especially large double‐stranded DNA (dsDNA) viruses such as vaccinia virus, herpes simplex virus (HSV), pseudorabies virus (PRV), cytomegalovirus, and ASFV manifest their pathogenicity through infection of terminally differentiated cells. During virus evolution, these viruses encode DNA synthesis enzymes involved in dNTP synthesis, such as ribonucleotide reductase (RNR), deoxyuridine 5′‐triphosphate nucleotidohydrolase (dUTPase), and thymidine kinase (TK).^[^ ^53^, ^54^ ^]^ When the expression of these genes is knocked out, dsDNA viruses are generally attenuated.^[^ ^41^, ^55^, ^56^ ^]^ Interestingly, some terminally differentiated cells, such as dendritic cells (DCs) and monocyte‐derived macrophages (MDMs), express a SAM domain and HD domain‐containing protein 1 (SAMHD1); this protein further depletes low intracellular dNTP pools, thereby limiting the replication of HIV‐1.^[^ ^57^ ^]^ In contrast, HIV‐2 and related simian immunodeficiency viruses (SIVsm/mac) replicate efficiently in these cells owing to their viral protein x (Vpx), which counteracts the SAMHD1 protein by mediating its degradation.^[^ ^57^, ^58^ ^]^ In addition, viral DNA synthesis can be attenuated in HIV‐2 and SIV pathogenic strains via Vpx gene modification.^[^ ^59^, ^60^ ^]^ Overall, modulation of viral RNA/DNA synthesis may be a useful strategy for designing potential LAVs.

### 2.4 Attenuation at the Translation Stage

Viral proteins exert their functions by translating viral messenger RNA (mRNA) into proteins, which decode the genetic information stored in viral mRNA. If this step is hijacked, the virus may also be attenuated. The poliovirus (PV) vaccine Sabin strains were generated through serial passaging of virulent PV in cultured cells, through which the virus progressively lost its neurovirulence.^[^ ^61^ ^]^ The attenuated phenotype was determined by mutations in the viral internal ribosome entry site (IRES), which translates genomic viral RNA via a cap‐independent mechanism. The IRES recruits eukaryotic initiation factor 4G (eIF4G) and polypyrimidine tract–binding protein (PTB) to translate viral mRNA, and mutations in the Sabin strains impair the binding of these proteins and thus reduce viral mRNA translation.^[^ ^61^ ^]^ Another example is the Newcastle disease virus (NDV), in which mutations in the methyltransferase motifs of large (L) polymerase proteins decrease viral pathogenicity through the modulation of viral translation.^[^ ^62^ ^]^ Furthermore, the double‐stranded RNA‐dependent protein kinase (PKR) in cells restricts viral replication through eIF2α‐mediated inhibition of viral RNA translation. Viruses have evolved a plethora of viral‐encoded antagonists to evade PKR restriction; thus, the absence of these antagonists in viruses leads to a decrease in their virulence.^[^ ^63^ ^]^

### 2.5 Attenuation by Restricting Cell‐to‐Cell Virus Spread

Viruses spread from infected cells to noninfected cells mainly through two distinct pathways. The first pathway involves the release of viral particles from infected cells, which subsequently attach to and enter new target cells. The second pathway is direct spread between contacting cells, also known as cell‐to‐cell spread.^[^ ^64^ ^]^ The ability of viral cell‐to‐cell transmission may also impact viral pathogenicity. In the case of a live attenuated CSFV vaccine, variations in the envelope 2 (E2) glycoprotein are responsible for viral attenuation by restricting the cell‐to‐cell spread of the virus.^[^ ^51^, ^65^ ^]^ The cell‐to‐cell fusion of SARS‐CoV‐2, mediated by the spike (S) protein, is intricately associated with its pathogenicity. The P681R mutation in the delta variant of SARS‐CoV‐2 has been shown to enhance both fusogenicity and pathogenicity.^[^ ^66^ ^]^ However, the fusogenicity of the Omicron S isoform was significantly impaired compared to that of the Delta spike, so the Omicron variant exhibited lower pathogenicity.^[^ ^67^ ^]^

Overall, while the mechanism of attenuation has been elucidated for certain LAVs, the attenuation mechanisms of the majority of LAVs are still unclear. Furthermore, attenuation may be linked to multiple stages of viral replication. Therefore, future research should prioritize the analysis of the attenuation mechanisms of successful LAVs.

## 3 Future Directions

Traditional LAVs are excellent in terms of efficacy and cost.^[^ ^4^, ^5^ ^]^ LAVs achieve remarkable success; however, they still pose certain challenges. First, LAVs are time‐consuming to develop and pose a danger to those with immunodeficiencies. Second, there is also a risk of pathogenic reversion. Third, their development relies heavily on empirical attenuation, which can be unreliable in some cases. We propose the following avenues for next‐generation LAVs.

### 3.1 Deciphering the Pathogenesis

To attenuate a specific virus, it is crucial to understand its pathogenicity toward the host and identify which genes contribute to its virulence. Consequently, we can modify virulence‐related genes while preserving immunogenicity genes, such as those of pseudorabies virus (PRV) and African swine fever virus (ASFV), which can be attenuated by knockout of expression of virulence genes.^[^ ^55^, ^68^ ^]^ Furthermore, DIVA (differentiating infected from vaccinated animals) vaccines hold promise for viral eradication, particularly in the treatment of veterinary diseases. The PRV LAV is the most successful DIVA vaccine for which expression of the viral thymidine kinase (TK) gene is knocked out to achieve attenuation and simultaneous knockout of glycoprotein E (gE) for differentiating infected animals from vaccinated animals.^[^ ^69^, ^70^ ^]^ Therefore, understanding viral pathogenesis and identifying the specific genes responsible for virulence are crucial in guiding the rational development of LAVs.

### 3.2 Directed Evolution of Viral Virulence‐Related Proteins

For some viruses, virulence‐related genes may be essential for viral replication and cannot be disrupted. To attenuate the virus while retaining its immunogenicity, rapid evolution of the selected virulence‐related gene may confer significant advantages. Directed evolution involves iterative cycles of mutation, amplification, and selection targeting genes of interest (GOIs).^[^ ^71^ ^]^ It is a powerful methodology for creating biomolecules with novel and improved properties and represents a pivotal aspect of modern biotechnology. In recent years, significant advancements and various artificial evolution methods have emerged in this field.^[^ ^71^, ^72^, ^73^, ^74^ ^]^ Therefore, rapid directed evolution may offer a promising strategy for expediting virus attenuation and accelerating the development of an optimal LAV. However, prior knowledge of the virulence‐determining genes discussed above is necessary for this method. Additionally, a robust screening platform is required to select an optimal evolved protein with an attenuated phenotype.

### 3.3 Novel Universal Technologies for Vaccine Development

The virulence‐related genes of some newly emerging viruses remain poorly characterized. In such scenarios, the application of universal technologies, such as codon pair bias (CPB) design, miRNA‐controlled LAVs, zinc‐finger nuclease‐controlled LAVs, targeted protein degradation (TPD) techniques, and premature termination codon (PTC) read‐through strategies, holds promise for addressing this issue.^[^ ^3^, ^6^, ^75^, ^76^ ^]^ CPB design is based on identifying genetic redundancy in the genetic code, allowing adjacent amino acids to be encoded by different pairs of synonymous codons. This design reveals that certain synonymous codon pairs are used more or less frequently than expected. In the case of poliovirus, this innovative approach was initially used to synthesize *de novo* DNA molecules encoding its capsid protein using multiple suboptimal synonymous codon pairs. This ultimately leads to the attenuation of polioviruses.^[^ ^75^ ^]^ CPB attenuation may involve diverse mechanisms: 1) a decrease in translation rate, 2) protein misfolding, 3) alterations in messenger RNA (mRNA) secondary structures, 4) modifications in regulatory signals, and 5) increased occurrence of dinucleotides.^[^ ^77^, ^78^ ^]^ This strategy exhibits potential for attenuating a broad spectrum of viruses, especially newly emerging viruses, such as SARS‐CoV‐2.^[^ ^79^, ^80^ ^]^ Moreover, CPB design confers another significant advantage over conventional LAVs; that is, extensive genetic modifications reduce the risk of virulence reversion.^[^ ^79^, ^80^ ^]^ However, this type of vaccine also has some drawbacks. The yield of modified viruses may be low, which restricts their large‐scale application because the purpose of this method is to reduce replication activity.^[^ ^75^ ^]^ Moreover, if the virus has not been extensively investigated, rescue attempts for the CPB virus might fail due to the destruction of an unidentified key element essential for viral replication. miRNA‐controlled LAVs, zinc‐finger nuclease‐controlled LAVs, TPD and PTC read‐through strategies have been thoroughly discussed in recent reviews.^[^ ^3^, ^6^ ^]^ TPD and PTC read‐through strategies to attenuate viruses include constructing conditionally replicating viruses (CRVs), a specific type of virus with impaired essential gene functions that disrupt viral genome replication, protein synthesis, or virus particle assembly.^[^ ^3^ ^]^ However, CRVs are characterized as single‐cycle infectious viruses and are not strictly classified as live viruses. Therefore, their efficacy may not be as robust as that of LAVs, and CRVs need multiple immunizations to achieve optimal results.^[^ ^76^ ^]^ Overall, novel universal technologies for vaccine development will pave a promising avenue for next‐generation LAVs.

## Conflict of Interest

The authors declare no conflict of interest.

## Author Contributions

All authors contributed equally to this work and are co‐corresponding authors for the review. All authors conceived, wrote, and discussed the manuscript.
